# Combination Immunotherapy with 4-1BBL and CTLA-4 Blockade for the Treatment of Prostate Cancer

**DOI:** 10.1155/2012/439235

**Published:** 2012-01-23

**Authors:** Kuang Youlin, Zhang Li, Weng Xiaodong, Liu Xiuheng, Zhu Hengchen

**Affiliations:** ^1^Renmin Hospital of Wuhan University, Wuhan 430060, China; ^2^Department of Urology, Renmin Hospital of Wuhan University, Jiefang Road 238, Wuhan 430060, China

## Abstract

Immune regulation has been shown to be involved in the progressive growth of some murine tumours. Interruption of immune regulatory pathways via activation of 4-1BB or cytotoxic T-lymphocyte-associated antigen-4 (CTLA-4) blockade appears to be a promising strategy for cancer immunotherapy. In this study, we examined the effectiveness of 4-1BBL-expressing tumor cell vaccine in combination with CTLA-4 blockade on rejection of murine prostate cancer RM-1. We found that the combination of both a vaccine consisting of 4-1BBL-expressing RM-1 cells and CTLA-4 blockade resulted in regression of RM-1 tumors and a significant increase in survival of the tumour cell recipients, compared to that of either treatment alone. The combined vaccination resulted in higher CTL against RM-1 cells and increased secretion of IFN-*γ*, TNF-*α, * and IL-2 in the mix-cultured supernatant. These results suggest that combining activation of 4-1BB and blockade of CTLA-4 may offer a new strategy for prostate cancer immunotherapy.

## 1. Introduction

Prostate cancer (PCa) is the most frequently diagnosed cancer in old men and also the second leading cause of male cancer death in the western countries [1]. In addition, the incidence and mortality of carcinoma of prostate are increasing in China. Although radical prostatectomy and radiation therapy remain the optimal choice for localized stage of PCa, there is no effective treatment for patients who develop recurrences or develop into hormone-resistance prostate cancer (HRPC) or those who have metastatic disease at the time of diagnosis. Therefore, new therapeutic approaches to control or even eliminate residual tumor cells are definitely needed, providing an opportunity for immunotherapy [2].

It is well known that T-cell-mediated immune response plays a great important role in antitumor immunity. An effective T-cell response can attack tumor cells only after T cell receives two key signals from the peptide/MHC complexes and costimulatory signals (including B7-1/2, 4-1BBL, and CD40). Without costimulation, T-cells will undergo apoptosis or become anergic [3–5]. The fact that tumor cells are found to have low expression of costimulatory molecule may explain how tumor cells evade the immune surveillance. Consistent with this possibility, researchers demonstrated that conferring 4-1BBL expression to tumors of a variety of tissue origins was, in many cases, sufficient to promote tumor rejection by a CD8^+^ T-cell-dependent mechanism [6, 7].

 4-1BBL (CD137L), the counterreceptor for 4-1BB, is a member of the TNF (ligand) superfamily and serves as a secondary signal to activated T cells. 4-1BB signaling can induce cytokine production, expansion, and functional maturation of T cells, dendritic cells, NK cells, and monocytes [8, 9]. With regard to tumor biology, binding of 4-1BB has been demonstrated to prevent and even rescue anergic CD8^+^ T cells in a number of tolerance-inducing models [10]. Also, 4-1BBL costimulation can retrieve CD28 expression in activated T cells [11]. A soluble 4-1BBL has also been shown to overcome immunological ignorance, allowing immunization with tumor-derived peptide to induce a protective CTL response [12]. 

 CTLA-4, a close homolog of CD28, is upregulated on activated T cells and binds B7-1 and B7-2 with considerably greater avidity than CD28 results in the transduction of an inhibitory signal and thereby functions as a negative regulator of T-cell activation in both CD4^+^ and CD8^+^ T cells [13]. When CTLA-4/B7 interactions are blocked by injection of anti-CTLA-4 monoclonal antibody during cancer vaccination, therapeutic T-cell immunity against even poorly immunogenic tumours such as B16 melanoma can be eliminated [14]. This effect is partly mediated by an increased expansion of antigen-specific CTL [15, 16]. It has been reported that blockade of CTLA-4/B7 interactions prevents induction of peripheral T-cell tolerance upon vaccination with peptides under tolerogenic conditions, suggesting that CTLA-4 might be actively involved in the induction of anergy [17].

 In the present work, we investigated the effect of a vaccine combined with 4-1BBL-expressing tumor vaccine and CTLA-4 blockade on the survival of C57BL/6 mice transplanted subcutaneously with prostate cancer RM-1 cells. We found that 4-1BBL-expressing tumor vaccine in combination with CTLA-4 blockade was effective in reducing tumor incidence and increasing in survival of the tumour cell recipients.

## 2. Materials and Methods

### 2.1. Animals, Cell Lines, and Antibodies

Female C57BL/6 (H-2 K^b^) mice, 6–8 weeks old, were obtained from Shanghai SLAC Laboratory Animal Co. Ltd (Shanghai, China). Animals were maintained at the Central Animal Facility of Wuhan University according to standard guidelines, and experiments were conducted according to the guidelines of the China Council for Animal Care. All mice are killed by cervical dislocation in the experiment. RM-1, a murine prostate cancer cell line, was obtained from Chinese Academy of Sciences (Shanghai, China). All cells were cultured in RPMI-1640 medium with 10% heat-inactivated FCS, 2 mM L-glutamine, 100 U/mL penicillin, and 100 *μ*g/mL streptomycin at 37°C in a humidified atmosphere containing 5% CO_2_. Anti-mouse CTLA-4 (clone 9H10) or hamster IgG isotype control was obtained from BioXCell; anti-4-1BBL was purchased from Santa Cruz.

### 2.2. Stable Transfection of RM-1 Cells with 4-1BBL Plasmid

The RM-1 cells were transfected with 2 *μ*g of pCDNA3.1-4-1BBL or empty vector by the mediation of 6 *μ*L Lipofectamine 2000 (Invitrogen, Carlsbad, CA, USA), according to the manufacturer's instructions. After 2 days of culture, the cells were reseeded into a 10 cm dish and cultured for another 2 days; complete RPMI-1640 medium containing 1000 *μ*g/mL G418 (Sigma, St. Louis, MO) was added to the culture. After 20 days of selection, all nontransfected cells died, and discrete clones were visible in transfected cells. These clones were expanded in the presence of 200 *μ*g/ml G418; positive cells expressing 4-1BBL or not were named RM-1/4-1BBL and RM-1/ pCDNA3.1.

### 2.3. Western Blot Analysis

To determine 4-1BBL expression, positive cells (5 × 10^6^) were lysed and subjected to SDS-PAGE. Then protein was transferred to a nitrocellulose membrane (Amersham, USA). The transferred membrane was probed with polyclonal goat anti-4-1BBL antibody, followed by a horseradish peroxidase-conjugated anti-goat IgG secondary antibody (Santa Cruz, CA). Antibodies on membrane were visualized by chemiluminescence (Pierce, Rockford, IL). Western blot for *β*-actin was used as an internal sample.

### 2.4. Subcutaneous Challenge and Immunization

Mice were shaved on the back and challenged subcutaneously with 2 × 10^5^ RM-1 cells in PBS. At the same day or later as indicated, parental and transduced cells were incubated with 100 *μ*g/mL mitomycin C (MMC) for 1 hour as cancer vaccine, and treatment was initiated by injecting 10^6^ cancer vaccine cells (in PBS) subcutaneously into the left flank and repeated 3 and 6 d later. Treatment with 9H10 or control hamster IgG was started simultaneously or 3 d later with similar results. Antibodies were delivered intraperitoneally at 100 mg in PBS, usually followed by two 50 mg injections every 3 d. Tumor growth was scored by measuring perpendicular diameters. Mice were killed when the tumors displayed severe ulceration or reached a size of 1000 mm^2^.

### 2.5. Generation of CTL Cultures and CTL Assay

Spleens were harvested from mice rejecting RM-1 cells and restimulated *in vitro* with MMC-treated RM-1 cells, and recombinant human IL-2 was added to a final concentration of 50 IU/mL. After 7 d, cells were collected and purified by Ficoll-Histopaque (Sigma-Aldrich) gradient centrifugation and served as effector cells. Target cells (2.5 × 10^5^ per well) were cocultured with effector cells (5 × 10^4^ per well) at different E : T ratios in 96 round bottom plates. After a 48-hour incubation at 37°C, the amount of released lactate dehydrogenase was determined by using Cell Counting Kit-8 (Dojindo, Japan) assay according to the manufacturer's instructions. All determinations were carried out in triplicate and repeated three times. The percentage of specific cytotoxicity was calculated as [target  control − (experimental − effectorcontrol)/target  control] × 100%.

### 2.6. Enzyme-Linked Immunosorbent Assay

24 h after target cell cocultured with effector cells, the supernatant was collected and tested for the presence of IFN-*γ*, TNF-*α*, and IL-2 by ELISA according to the manufacturer's instructions (Pharmingen).

### 2.7. Statistical Analysis

Data were presented as mean ± standard deviation. Statistical differences were considered to be significant at a Pvalue <0.05 as determined by an ANOVA or Student's *t*-test using SPSS13.0. Comparison among groups in the survival data was made using the log-rank test.

## 3. Results

### 3.1. Establishment of RM-1 Cells Expressing 4-1BBL

**Figure 1:**
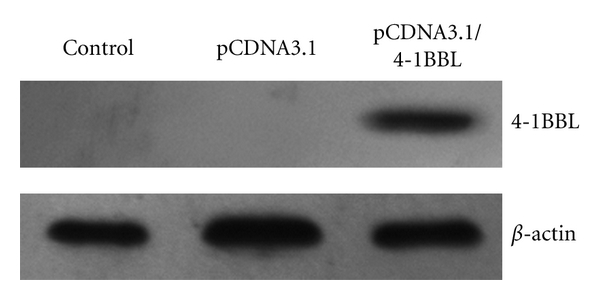
Total cell lysates were harvested, and presence of 4-1BBL protein was detected by anti-4-1BBL pAb. A specific band was identified in positive clone but not in RM-1 cells transfected with empty vector. *β*-actin was used as reference.

RM-1 cells were transfected with pCDNA3.1 and pCDNA3.1/4-1BBL, and the G418-resistant cells (RM-1/pCDNA3.1, RM-1/4-1BBL) were selected. Western blot analysis showed the expression of 4-1BBL in parental and transduced cells, respectively (Figure 1).

### 3.2. CTLA-4 Blockade Together with 4-1BBL-Expressing Cellular Vaccines Causes Rejection of RM-1 Tumors

**Figure 2:**
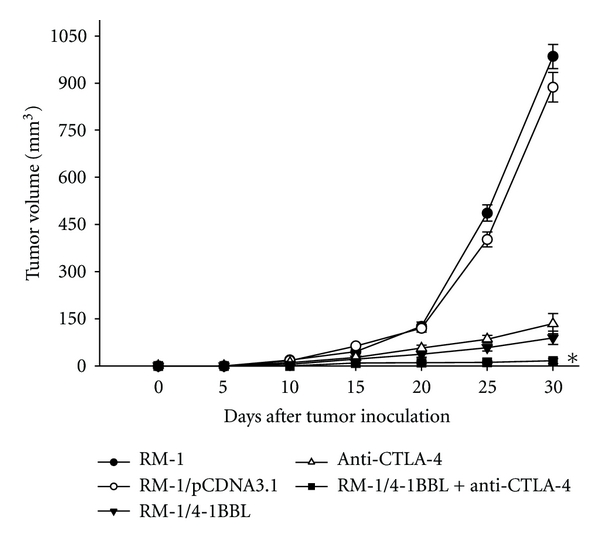
Antitumor immunity *in vivo*. Mice were challenged subcutaneously with RM-1 cells (2 × 10^5^ cells/mouse), then 10^6^ tumor vaccine cells (RM-1, RM-1/pCDNA3.1, RM-1/4-1BBL) were immunized at the same day, with 9H10 or control hamster IgG intraperitoneally every 3 d. The data are expressed as means ± SD of three replicates (*P < 0.05).

To determine the effect of 4-1BBL-expressing cellular vaccines combined with CTLA-4 blockade on tumor growth in vivo, parental RM-1, RM-1/pCDNA3.1, and pCDNA3.1/4-1BBL cells were injected into the flank of mice. Tumor growth in mice injected with pCDNA3.1/4-1BBL cells was slower than that in mice injected with parental RM-1 or RM-1/pCDNA3.1. Administration of anti-CTLA-4 antibody 9H10 delayed growth of RM-1 tumors, but control hamster IgG had no effect. However, the combination of 4-1BBL-expressing vaccine and CTLA-4 blockade induced more obvious effectiveness on RM-1 tumor growth than either treatment alone. 4/5 mice rejected RM-1 tumors after the combinated treatment (Figure 2).

### 3.3. CTLA-4 Blockade Together with 4-1BBL-Expressing Cellular Vaccines Increased CTL Activity and Production of Cytokines

**Figure 3:**
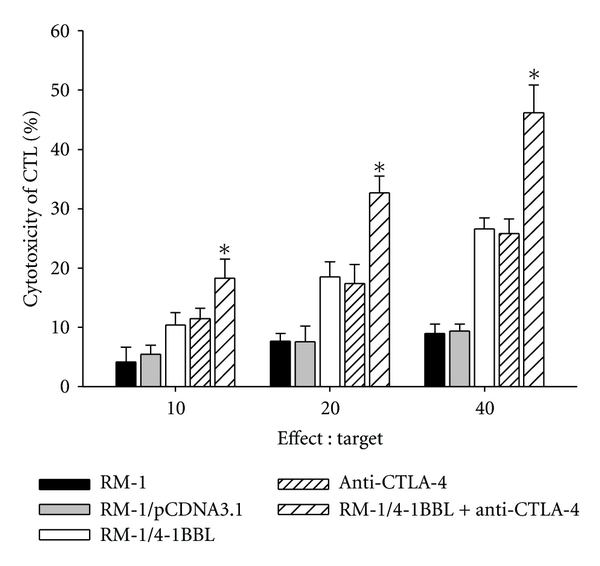
Cytotoxicity assay. Spleens were harvested from mice rejecting RM-1 cells and restimulated in vitro with MMC-treated RM-1 cells. 7 d later, cells were collected and purified by Ficoll-Histopaque gradient centrifugation as effector cells for detecting specific cytotoxicity against target cells. The data are expressed as means ± SD of three replicates (*P < 0.05).

**Figure 4:**
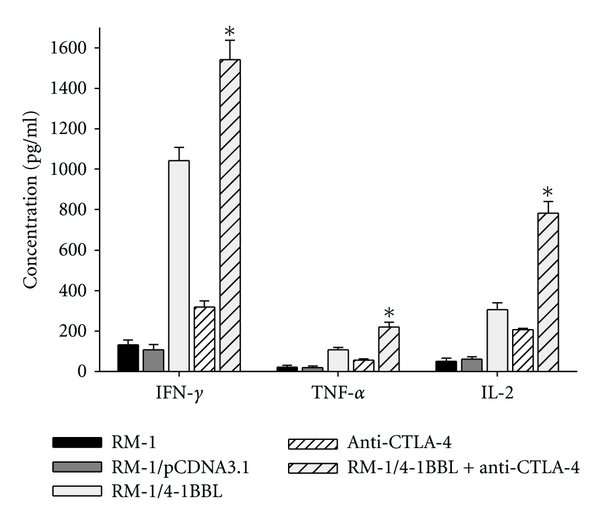
Cytokine ELISA. Spleens were harvested from mice rejecting RM-1 cells and restimulated in vitro with MMC-treated RM-1 cells. 7 d later, cells were collected and purified by Ficoll-Histopaque gradient centrifugation as effector cells coculturing with RM-1 cells. The The supernatant was collected and tested for the presence of IFN-*γ*, TNF-*α* and IL-2 by ELISA (*P < 0.05).

To determine the immune function of 4-1BBL-expressing cellular vaccines combined with CTLA-4 blockade in vitro, CTL activity of splenocytes from immunized mice was evaluated. As shown in Figure 3, CTL activity of splenocytes from mice immunized with the combination of 4-1BBL-expressing vaccine and CTLA-4 blockade was dramatically higher than that from mice treated either alone. Vaccination with parental RM-1, RM-1/pCDNA3.1, and control hamster IgG had no effect on CTL activity. Also, the level of cytokines in supernatant cocultured cells was examined. The results showed that the levels of cytokines (IFN-*γ*, TNF-*α*, and IL-2) in supernatant from mice vaccinated with 4-1BBL-expressing vaccine and CTLA-4 blockade were much higher than that from mice immunized with either alone (Figure 4).

### 3.4. CTLA-4 Blockade Together with 4-1BBL-Expressing Cellular Vaccines Prolonged the Life Span of Mice Rechallenged Tumors

**Figure 5:**
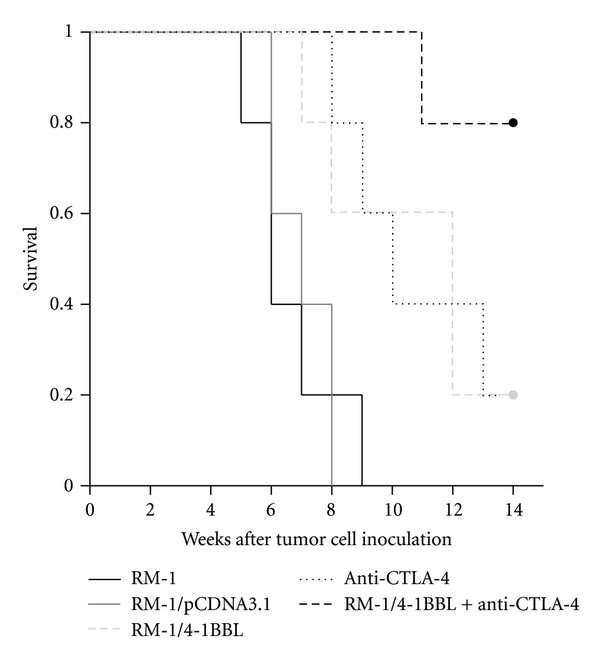
Kaplan-Meier survival curve of mice. According to the log-rank test, there were significant differences among five groups (*P* < 0.05). Compared with those of the other two groups, the survival rate of mice immunized with 4-1BBL-expressing cellular vaccines combined with CTLA-4 blockade was significant higher than those of mice immunized with either alone (*P* < 0.05).

To determine the immune protection effect of 4-1BBL-expressing cellular vaccines combined with CTLA-4 blockade, mice were inoculated subcutaneously with lethal dose of parental RM-1 cells to monitor survival daily. The survival rate of mice immunized with both CTLA-4 blockade and 4-1BBL-expressing cellular vaccines was significantly higher than that of mice immunized with either alone (Figure 5).

## 4. Discussion

In the present study, we demonstrated that the preclinical effect of 4-1BBL-expressing cellular vaccines can be markedly improved by combining vaccination with treatment with anti-CTLA-4 mAb. The combination vaccine resulted in sustained tumor degradation in all the mice. Our treatment regimen holds great promise for a positive clinical effect in humans.

 Actually, cancer occurrence and development has been demonstrated to be associated with escape from immune surveillance. The prostate cancer vaccine alone was unable to cause complete tumor regression, which could reflect either that the initial CD8^+^ T-cell-mediated antitumor immune response simply is not potent enough to completely eliminate all the cancer cells or that the cancer cells have lost their immunogenicity. There might be several reasons for that including (1) low-level expression of the major MHC molecules, (2) absence of recognized tumor Ags, (3) poor costimulatory molecule expression, or (4) some kind of immunosuppression of the CD8^+^ T-cell response such as TGF-*β* [18]. 4-1BB is an inducible member of the TNFR superfamily that has profound effects on T cells, including activation of both CD4^+^ and CD8^+^ T cells, enhanced expansion [19, 20], increased long-term survival [21, 22], and antiapoptosis of activation-induced CD8^+^ T cells [23]. Costimulation through 4-1BB can also promote enhanced production of cytokines such as IL-2, IL-4, and IFN-*γ* [19, 24]. Regarding this, we sought to understand the effect on tumor-specific T cell responses of simultaneously actively driving proliferation and survival through activation of the costimulatory receptor 4-1BB, while at the same time eliminating a major brake on expansion via blocking the coinhibitory receptor CTLA-4.

 In murine experiments, activation of 4-1BB with 4-1BB mAb can lead to rejection of many tumours [25]. Indeed, phase I and II clinical trials using anti-4-1BB therapy for advanced cancers are underway [26]. However, anti-4-1BB antibodies can cause severe immune system anomalies when given systemically [27]. Thus, in the present study, we use 4-1BBL-expressing cellular vaccines for treatment of prostate cancer. Our data indicate that the 4-1BBL-expressing cellular vaccine did slow tumor growth when initiated at the time of tumor implantation and resulted in regression of tumors in about 2/5 of the mice. Moreover, the combination of 4-1BBL-expressing cellular vaccine with CTLA-4 blockade induced rejection of all tumors injected at the same day. These results suggest that 4-1BBL-expressing cellular vaccines can be markedly improved by combining vaccination with treatment with anti-CTLA-4 mAb. In the study, we demonstrated the number of CD8^+^ and CD4^+^ T cells in RM-1 tumors in mice immunized with anti-CTLA-4 mAb and 4-1BBL-expressing cellular vaccines (data not shown), which suggest enhancement of cytotoxicity of TIL might be a way for the combination of vaccine to execute antitumor effect. The same results were observed in B16 melanoma by Kocak et al. [28] and Curran et al. [29].

 Our results demonstrated that CTL activity of splenocytes and cytokine from mice immunized with 4-1BBL-expressing cellular vaccines and CTLA-4 blockade was dramatically increased compared with that from mice immunized with either alone. Vaccination with parental RM-1, RM-1/pCDNA3.1, and control hamster IgG had no effect on CTL activity. The main effector cells performing CTL activity are CD8^+^ T cells, while the main cells producing cytokines such as IL-2, IFN-*γ*, and TNF-*α* are CD4^+^ Th1 cells. Th1 cells play a critical role in cellular immunity by their cytokines activating CD8^+^ T cells. Long-term survival of mice immunized with 4-1BBL-expressing cellular vaccines and CTLA-4 blockade when rechallenged lethal dose of parental RM-1 cells indicated that the combination of vaccines executes antitumor immune response by activating CD8^+^ and CD4^+^ T cells.

The findings presented in this study have significant implications for immunotherapy in humans. Our results suggested that it is important to consider whether two treatments will act synergistically when developing an immunotherapeutic strategy. Moreover, they also suggest that CTLA-4 blockade may be a vital adjuvant for a 4-1BBL-expressing vaccine used to treat cancers.

## 5. Conclusion

 In summary, we concluded that the combination of activation of 4-1BB and blockade of CTLA-4 has a higher potential antitumor effect and may offer a new strategy for prostate cancer immunotherapy.

## References

[1] Jemal A, Siegel R, Ward E (2008). Cancer statistics, 2008. *CA Cancer Journal for Clinicians*.

[2] Aldrich JF, Lowe DB, Shearer MH, Winn RE, Jumper CA, Kennedy RC (2010). Vaccines and immunotherapeutics for the treatment of malignant disease. *Clinical and Developmental Immunology*.

[3] Guinn BA, Bertram EM, DeBenedette MA, Berinstein NL, Watts TH (2001). 4-1BBL enhances anti-tumor responses in the presence or absence of CD28 but CD28 is required for protective immunity against parental tumors. *Cellular Immunology*.

[4] Habib-Agahi M, Phan TT, Searle PF (2007). Co-stimulation with 4-1BB ligand allows extended T-cell proliferation, synergizes with CD80/CD86 and can reactivate anergic T cells. *International Immunology*.

[5] Breckpot K, Escors D (2009). Dendritic cells for active anti-cancer immunotherapy: targeting activation pathways through genetic modification. *Endocrine, Metabolic and Immune Disorders—Drug Targets*.

[6] Li Q, Ai J, Song Z, Liu J, Shan B (2008). 4-1BB (CD137) ligand enhanced anti-tumor immune response against mouse forestomach carcinoma in vivo. *Cellular & Molecular Immunology*.

[7] Yan X, Johnson BD, Orentas RJ (2008). Induction of a VLA-2 (CD49b)-expressing effector T cell population by a cell-based neuroblastoma vaccine expressing CD137L. *Journal of Immunology*.

[8] Wilcox RA, Tamada K, Strome SE, Chen L (2002). Signaling through NK cell-associated CD137 promotes both helper function for CD8^+^ cytolytic T cells and responsiveness to IL-2 but not cytolytic activity. *Journal of Immunology*.

[9] Cannons JL, Lau P, Ghumman B (2001). 4-1BB ligand induces cell division, sustains survival, and enhances effector function of CD4 and CD8 T cells with similar efficacy. *Journal of Immunology*.

[10] Wilcox RA, Tamada K, Flies DB (2004). Ligation of CD137 receptor prevents and reverses established anergy of CD8^+^ cytolytic T lymphocytes in vivo. *Blood*.

[11] Habib-Agahi M, Jaberipour M, Searle PF (2009). 4-1BBL costimulation retrieves CD28 expression in activated T cells. *Cellular Immunology*.

[12] Sharma RK, Elpek KG, Yolcu ES (2009). Costimulation as a platform for the development of vaccines: a peptide-based vaccine containing a novel form of 4-1BB ligand eradicates established tumors. *Cancer Research*.

[13] Hodi FS (2007). Cytotoxic T-lymphocyte-associated antigen-4. *Clinical Cancer Research*.

[14] van Elsas A, Hurwitz AA, Allison JP (1999). Combination immunotherapy of B16 melanoma using anti-cytotoxic T lymphocyte-associated antigen 4 (CTLA-4) and granulocyte/macrophage colony- stimulating factor (GM-CSF)-producing vaccines induces rejection of subcutaneous and metastatic tumors accompanied by autoimmune depigmentation. *Journal of Experimental Medicine*.

[15] Met O, Wang M, Pedersen AE, Nissen MH, Buus S, Claesson MH (2006). The effect of a therapeutic dendritic cell-based cancer vaccination depends on the blockage of CTLA-4 signaling. *Cancer Letters*.

[16] Sotomayor EM, Borrello I, Tubb E, Allison JP, Levitsky HI (1999). In vivo blockade of CTLA-4 enhances the priming of responsive T cells but fails to prevent the induction of tumor antigen-specific tolerance. *Proceedings of the National Academy of Sciences of the United States of America*.

[17] Zhang F, Huang G, Hu B, Song Y, Shi Y (2011). Induction of immune tolerance in asthmatic mice by vaccination with DNA encoding an allergen-cytotoxic T lymphocyte-associated antigen 4 combination. *Clinical and Vaccine Immunology*.

[18] Elkord E (2007). Immunology and immunotherapy approaches for prostate cancer. *Prostate Cancer and Prostatic Diseases*.

[19] Lu ZY, Condomines M, Tarte K (2007). B7-1 and 4-1BB ligand expression on a myeloma cell line makes it possible to expand autologous tumor-specific cytotoxic T cells in vitro. *Experimental Hematology*.

[20] Laderach D, Movassagh M, Johnson A, Mittler RS, Galy A (2002). 4-1BB co-stimulation enhances human CD8^+^ T cell priming by augmenting the proliferation and survival of effector CD8^+^ T cells. *International Immunology*.

[21] Lee HW, Park SJ, Choi BK, Kim HH, Nam KO, Kwon BS (2002). 4-1BB promotes the survival of CD8^+^ T lymphocytes by increasing expression of Bcl-xL and Bfl-1. *Journal of Immunology*.

[22] Croft M (2003). Co-stimulatory members of the TNFR family: keys to effective T-cell immunity?. *Nature Reviews Immunology*.

[23] Kudo-Saito C, Hodge JW, Kwak H, Kim-Schulze S, Schlom J, Kaufman HL (2006). 4-1BB ligand enhances tumor-specific immunity of poxvirus vaccines. *Vaccine*.

[24] Xiao H, Huang B, Yuan Y (2007). Soluble PD-1 facilitates 4-1BBL—Triggered antitumor immunity against murine H22 hepatocarcinoma in vivo. *Clinical Cancer Research*.

[25] Li Q, Iuchi T, Jure-Kunkel MN, Chang AE (2007). Adjuvant effect of anti-4-1BB mAb administration in adoptive T cell therapy of cancer. *International Journal of Biological Sciences*.

[26] Lynch DH (2008). The promise of 4-1BB (CD137)-mediated immunomodulation and the immunotherapy of cancer. *Immunological Reviews*.

[27] Wang C, Lin GHY, McPherson AJ, Watts TH (2009). Immune regulation by 4-1BB and 4-1BBL: complexities and challenges. *Immunological Reviews*.

[28] Kocak E, Lute K, Chang X (2006). Combination therapy with anti-CTL antigen-4 and anti-4-1BB antibodies enhances cancer immunity and reduces autoimmunity. *Cancer Research*.

[29] Curran MA, Kim M, Montalvo W, Al-Shamkhani A, Allison JP (2011). Combination CTLA-4 blockade and 4-1BB activation enhances tumor rejection by increasing T-cell infiltration, proliferation, and cytokine production. *PLoS ONE*.

